# The Presence of Modifiable Residues in the Core Peptide Part of Precursor Nisin Is Not Crucial for Precursor Nisin Interactions with NisB- and NisC

**DOI:** 10.1371/journal.pone.0074890

**Published:** 2013-09-09

**Authors:** Rustem Khusainov, Oscar P. Kuipers

**Affiliations:** 1 Molecular Genetics Department, University of Groningen, Groningen, The Netherlands; 2 Kluyver Centre for Genomics of Industrial Fermentation, Groningen, The Netherlands; 3 Synthetic Biology Centre, University of Groningen, Groningen, The Netherlands; Griffith University, Australia

## Abstract

Precursor nisin is a model posttranslationally modified precursor lantibiotic that can be structurally divided into a leader peptide sequence and a modifiable core peptide part. The nisin core peptide clearly plays an important role in the precursor nisin – nisin modification enzymes interactions, since it has previously been shown that the construct containing only the nisin leader sequence is not sufficient to pull-down the nisin modification enzymes NisB and NisC. Serines and threonines in the core peptide part are the residues that NisB specifically dehydrates, and cysteines are the residues that NisC stereospecifically couples to the dehydrated amino acids. Here, we demonstrate that increasing the number of negatively charged residues in the core peptide part of precursor nisin, which are absent in wild-type nisin, does not abolish binding of precursor nisin to the modification enzymes NisB and NisC, but dramatically decreases the antimicrobial potency of these nisin mutants. An unnatural precursor nisin variant lacking all serines and threonines in the core peptide part and an unnatural precursor nisin variant lacking all cysteines in the core peptide part still bind the nisin modification enzymes NisB and NisC, suggesting that these residues are not essential for direct interactions with the nisin modification enzymes NisB and NisC. These results are important for lantibiotic engineering studies.

## Introduction

Nisin is a prominent lantibiotic produced by some strains of *Lactococcus lactis.* Lantibiotics form a class of ribosomally synthesized antimicrobial peptides, highly active against Gram-positive bacteria. The spread of multi-drug resistant bacteria is an alarming issue, especially in view of the decreasing effectiveness of conventional antibiotics. To tackle this problem, novel antimicrobial agents are urgently needed. Lantibiotics are very promising candidates to replace conventional antibiotics. Their unique characteristics include the presence of lanthionine and (methyl)lanthionine rings and a high stability upon heat or proteolytic treatment [1], [2]. Precursor nisin consists of an N-terminal leader peptide sequence and a C-terminal modifiable core peptide part. Dehydrated residues are introduced posttranslationally by NisB, specifically into the core peptide part, through dehydration of serines and threonines, yielding Dha (dehydroalanine) and Dhb (dehydrobutyrine), respectively. Lanthionine and (methyl)lanthionine rings are formed by intramolecular coupling of cysteines to the dehydrated residues in a stereospecific manner by the cyclase NisC. Fully modified precursor nisin is transported by a dedicated ABC transporter, NisT, to the outside of the cell, where the protease NisP cleaves off the leader, liberating active nisin [3], [4]. Recently, a complex of nisin modification enzymes has been isolated, consisting of NisB, NisC and NisT [5].

Dehydration and cyclization processes for class I and class II lantibiotics have been shown to proceed from the N-terminal to the C-terminal end [6], [7] and an alternating mode of action between NisB and NisC has been proposed [6], [8]. Interestingly, in contrast to class I and II lantibiotics, class III lantibiotics synthetase LabKC has a C- to N-terminus processing mode [9].

Nisin exerts at least two modes of antimicrobial action: i) by binding to lipid II, an essential intermediate for cell wall biosynthesis, it inhibits cell wall synthesis [10], [11]; and ii) by forming pores in the cell membrane, it releases cellular constituents [11]. Nuclear magnetic resonance studies performed with nisin and lipid II in sodium dodecyl sulfate micelles demonstrated that the rings A and B of nisin are important for the nisin-lipid II interactions [12]. In particular, formation of two intermolecular hydrogen bonds between Dhb2 (dehydrobutyrine) and Abu8 (α-aminobutyric acid) of nisin and the pyrophosphate moiety of a lipid II analog was demonstrated experimentally [13]. Moreover, it was shown that the formation of intermolecular hydrogen bonds and contact of nisin with the electronegative pyrophosphates of lipid II triggers the formation of a cage-like structure, consisting of rings A and B [13]. Due to sequestering of lipid II, nisin is active at nM concentrations. Nisin additionally inhibits the outgrowth of spores via lipid II-dependent pore formation [14]. Class I lantibiotics possess two modification enzymes, i.e. a dehydratase LanB and a cyclase LanC. Class II lantibiotics are modified by a bi-functional enzyme called LanM, which is able to perform both the dehydration and the cyclization reactions. The C-terminal part of LanM enzymes has a low sequence similarity to that of LanC enzymes and is anticipated to perform the cyclization reaction. However, the N-terminal part of LanM does not share any homology with NisB. Applying various unnatural substrates, it has been demonstrated that the class I NisBTC machinery as well as the class II LanM enzymes can modify non-lantibiotic peptides, if they N-terminally possess the class I nisin leader or a class II leader [15]–[21]. Moreover, the class II LctM enzyme has been shown to modify peptides where the core peptide and the leader peptide parts are separated by several alanine residues and more excitingly, peptides that had non-proteinogenic amino acids in the leader or in the core peptide parts were also modified [18]. This suggests that lantibiotic modification enzymes have very relaxed substrate specificities.

The nisin leader is an important site of interaction between precursor nisin and the modification enzymes NisB and NisC [5], [22]–[25]. Furthermore, the class I and class II lantibiotics have been observed to be (partially) modified in the absence of the leader [20], [22]. However, the core peptide part has also been demonstrated to be involved in the interactions with NisB and NisC [22]. This notion is important in the light of increased interest to engineering of lantibiotics. What particular residues in the core peptide are involved in the interactions with the nisin modification enzymes NisB and NisC is not known.

Successful examples of the enhancement of the antimicrobial activity of nisin by protein engineering exist [26], [27]. Ring engineering studies of nisin have demonstrated that to exert antimicrobial activity, the thioether rings ABC of nisin should be present [28]. Opening of ring A results in significant loss of antimicrobial activity against *Micrococcus luteus* NCDO 8166 and leads to a complete loss of antimicrobial activity against *Lactococcus lactis* MG1614 [28]. Opening of ring B results in a lack of antimicrobial activity against *L. lactis* LL108 [29]. Ring C has been shown to be essential for nisin antimicrobial activity by a) thermolysin cleavage of the Ala15-Leu16 and subsequently the Leu16-Met17 bonds in ring C of nisin(1–29); b) introducing a tryptic cleavage site into ring C by substituting the Met17 by Lys and subsequent trypsin treatment [30]; c) engineering a disulfide bond instead of a lanthionine that, upon reduction, caused a total loss of activity [31].

Negatively charged amino acids are not naturally found in the core peptide part of precursor nisin and closely related peptides such as subtilin and epidermin [32]. The absence of negatively charged amino acids in the core peptide region of precursor nisin was intriguing to us and might be an indication that negatively charged residues are unfavourable for the interactions with the nisin modification machinery and/or for modification. Another possibility is that negatively charged residues have a negative impact on the antimicrobial activity of nisin. Which exact residues in precursor nisin are involved in the interactions with the nisin modification enzymes NisB and NisC is not known. The development of a nisin binding assay [5] facilitates molecular characterization of the interactions between precursor nisin and its modification enzymes [5]. In this study, we perform co-purification studies of the nisin modification enzymes NisB and NisC with structurally unnatural precursor nisin molecules lacking modifiable residues in the core peptide part as well as with mutants that have various numbers of negatively charged residues in the core peptide part, to investigate whether binding of the modifying enzymes to precursor nisin and the substrate-enzyme complex formation are hampered, and whether modifications can still occur.

## Materials and Methods

### Synthetic nisin variants

Synthetic DNA encoding nisin variants was ordered from Life Technologies and subsequently cloned into a pNZnisAE3 vector [15] through cutting out the *nisA* gene by utilizing BglII and HindIII restriction sites, and cloning of the synthetic DNA, encoding precursor nisin, using the same restriction sites.

### Recombinant DNA techniques

Standard genetic manipulations were essentially performed as described by Sambrook *et al.* [33]. Plasmid isolation was performed by means of the Plasmid DNA Isolation Kit (Roche Applied Science). Restriction analysis was performed with restriction enzymes from Fermentas. DNA ligation was performed with T4 DNA ligase (Fermentas) and round PCR amplification was done with Phusion DNA polymerase (Finnzymes).

### Bacterial strains and growth conditions

Strain *Lactococcus lactis* NZ9000 [34] was used as an expression host in this study. In short, cells were grown as described previously [5] at 30°C in M17 medium (Difco) supplemented with 0.5% (w/v) glucose and 5 µg/ml chloramphenicol or 5 µg/ml erythromycin where appropriate. In case both antibiotics were used simultaneously, 4 µg/ml chloramphenicol and 4 µg/ml erythromycin were applied.

### Antimicrobial activity assay

The indicator strain *L.lactis* NZ9000 bearing plasmid pNZnisPT was grown ON in M17 medium supplemented with 0.5% glucose. Next morning it was re-inoculated into a fresh M17 medium containing 0.5 ng/ml nisin for induction. When the OD_600_ reached 0.6, 100 µl of the culture was added to 100 ml of liquid M17 agar at 40°C. Plates were dried, and wells were made. 50 µl Ni-NTA purified samples were applied to the wells and the plates were left ON at 30°C.

### Ni-NTA purification

Ni-NTA purification was performed as previously described [5]. In brief, 1.5 ml of 50% superflow Ni-NTA column resin (Qiagen) was equilibrated twice with 38.5 ml lysis buffer (50 mM NaH_2_PO_4_, 300 mM NaCl, 10 mM imidazole, pH 8) in a 50 ml tube by mixing on a rotor for 30 minutes. Subsequently, column material was resuspended in a 4–8 ml cytoplasmic fraction, transferred into a 15 ml tube, lysis buffer was added to a final volume of 12 ml and histagged protein was allowed to bind to the column material on a rotor in the cold room at 4°C for 2 hours. Subsequently, the column was washed twice with 35 ml of wash buffer (50 mM NaH_2_PO_4_, 300 mM NaCl, 20 mM imidazole, pH 8). Elutions were collected in four fractions of 0.5 ml each with elution buffer (50 mM NaH_2_PO_4_, 300 mM NaCl, 250 mM imidazole, pH 8). Fractions were analyzed by SDS-PAGE and Western Blot.

### SDS-PAGE and Western Blot

Western blots were performed using anti-NisB, anti-NisC or anti-leader antibodies [5]. SDS-PAGE was done using standard molecular biology techniques [33]. Samples were not boiled before applying to SDS-PAGE.

## Results

**Figure 1:**
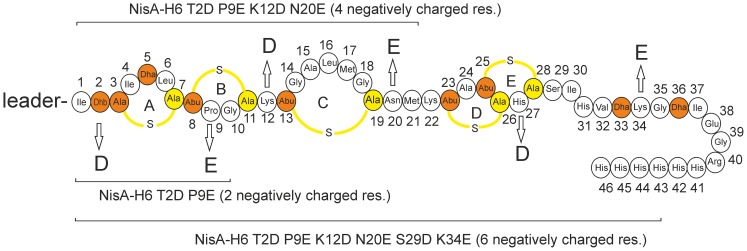
Schematic drawing of precursor nisin NisA-H_6_ mutants with increasing numbers of negatively charged residues. (The arrows indicate the residues that were substituted either by D (Asp) or E (Glu). Dehydrated Ser/Thr are depicted in orange, cysteines that that are coupled to dehydroresidues are depicted in yellow.

In order to investigate the substrate specificity of the nisin modification enzymes and to determine the binding requirements of precursor nisin to the modification enzymes NisB and NisC, we engineered two synthetic nisin variants, *i.e.* one lacking all serine and threonine residues in the core peptide part, and a second one lacking all cysteines. Additionally, precursor nisin variants with increasing numbers of negatively charged amino acids were made (Fig. 1; Table 1). A previously described prenisin interaction assay was used that allows co-purification of the nisin modification enzymes NisB and NisC using a precursor nisin with a C-terminal His-tag extension as bait [5].

**Table 1:** *Lactococcus lactis* strains and plasmids used in this study.

Strain	Characteristics	References
NZ9000	*nisRK*	[34]
Plasmids		
pNZnisA-E3	*nisA*	[15]
pNZnisA-H_6_	*nisA*, encoding precursor nisin NisA-H_6_, C-terminally extended with codons for GSIEGRHHHHHH	[5]
pIL32BTC	*nisBTC*, encoding nisin modification machinery	this study
pNZnisA-H_6_ T2D P9D	*nisA* encoding NisA-H_6_ with T2D P9D substitutions	this study
pNZnisA-H_6_ T2D P9D K12D N20E	*nisA* encoding NisA-H_6_ with T2D P9D K12D N20E substitutions	this study
pNZnisA-H_6_ T2D P9D K12D N20E H27D K34E	*nisA* encoding NisA-H_6_ with T2D P9D K12D N20E H27D K34E substitutions	this study
pNZnisA-H_6_ T2A S3A S5A 8TA T13A T23A T25A S36A	*nisA* encoding NisA-H_6_ with T2A S3A S5A 8TA T13A T23A T25A S36A substitutions (Ser/Thr-less)	this study
pNZnisA-H_6_ C7A C11A C19A C26A C28A	*nisA* encoding NisA-H_6_ with C7A C11A C19A C26A C28A substitutions (Cys-less)	this study

### Ser/Thr-less and Cys-less variants of precursor nisin still bind NisB and NisC

**Figure 2:**
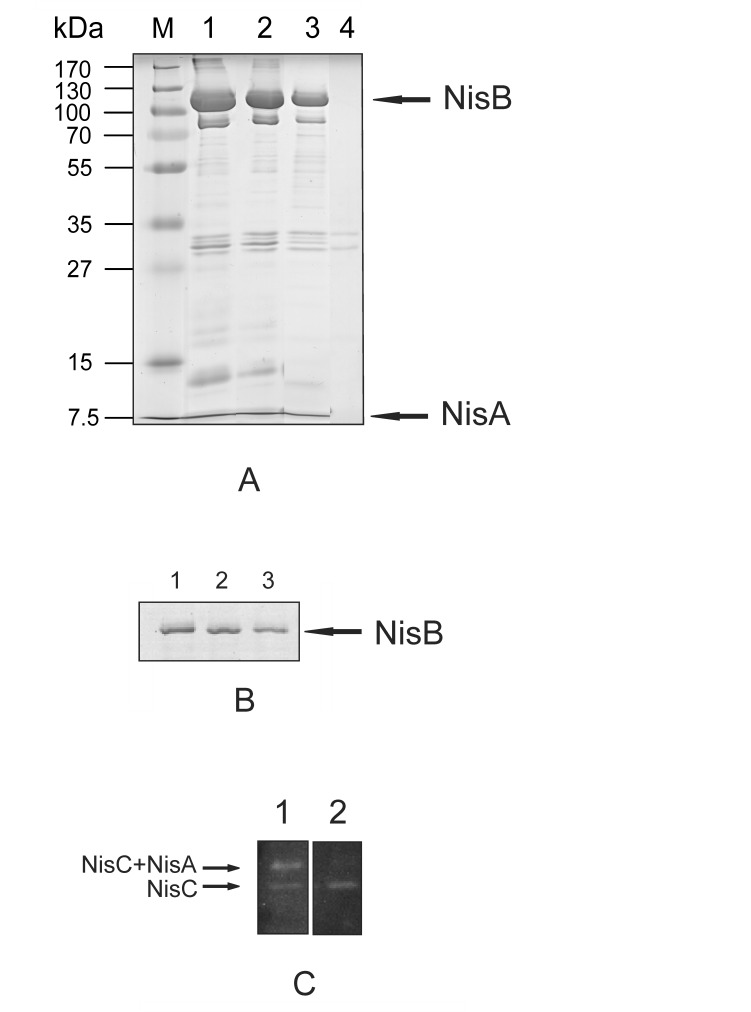
Co-purification assay of the nisin modification enzymes NisB and NisC with different precursor nisin variants. 2A. SDS-PAGE analysis of co-purification assay of strains expressing wild type enzymes NisBTC and: *Lane 1:* precursor nisin NisA-H6; *Lane 2:* Cys-less precursor nisin (NisA-H_6_ C-less); *lane 3*: Ser/Thr-less precursor nisin (NisA-H_6_-TS); *Lane 4:* empty vector (negative control). 2B. SDS-PAGE analysis of co-purification assay of strains expressing wild type enzymes NisBTC and: *Lane 1*: NisA-H_6_ T2D P9E; *lane 2:* NisA-H_6_ T2D P9E K12D N20E; *lane 3:* NisA-H_6_ T2D P9E K12D N20E H27D K34E. 2C. Western blot of co-purification assay of strains expressing wild type enzymes NisBTC and precursor nisin variants. Co-purified NisC was detected by anti-NisC antibodies. *Lane 1*: Cys-less precursor nisin (NisA-H_6_ C-less); *lane 2*: Ser/Thr- less precursor nisin (NisA-H_6_ TS-less). The lower band represents NisC, the upper band represents NisC in a complex with NisA.

Unnatural precursor nisin variants, one lacking all serines/threonines and another one lacking all cysteines (all these residues were replaced by Ala residues) were expressed (Fig. 1) and employed as substrates for *in vivo* modification. Both synthesized variants were still able to bind NisB and NisC and able to pull them down (Fig. 2A and 2C). This observation indicates that the presence of serines and threonines in the core peptide part of precursor nisin is not essential for NisB binding, and the presence of cysteines is not absolutely necessary for NisB or NisC binding. However, in the situation with Ser/Thr-less precursor nisin, only NisC can be detected (Fig. 2C, lane 2) but not the complex of NisB-NisA, as detected with Cys-less precursor nisin (Fig. 2C, lane 1).

### Negatively charged residues in the core peptide do not influence NisB and NisC interactions

The requirements for binding of the nisin modification enzymes NisB and NisC to precursor nisin are currently unknown. The leader peptide is crucially important, but electrostatic interactions with the core peptide might also have an influence. To investigate this hypothesis, we introduced additional negative charges into the core peptide part of precursor nisin (Fig. 1). Three variants of precursor nisin with an increasing number of negatively charged residues were made, namely NisA-H_6_ T2D P9D with two negatively charged residues, NisA-H_6_ T2D P9D K12D N20E with four negatively charged residues and NisA-H_6_ T2D P9D K12D N20E H27D K34E with six negatively charged residues (Fig. 1). These precursor nisin mutants were able to co-purify with NisB, indicating binding to NisB (Fig. 2B).

### Effect of negatively charged residues in the core peptide on the antimicrobial activity of precursor nisin

**Figure 3:**
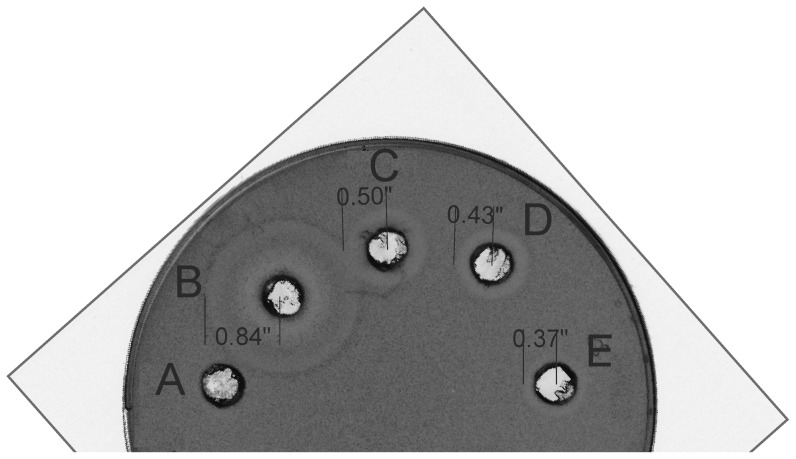
Antimicrobial activity assay of NisA-H6 variants purified from strains expressing wild type enzymes NisBTC. 50 µl of Ni-NTA purified samples were applied to the wells in the agar plate containing nisin sensitive indicator strain *L.lactis* NZ9000 expressing NisPT. Well A: empty plasmid pNZ8048E (negative control); well B: wild type NisA-H_6_; well C: NisA-H6 T2D P9D with two negatively charged residues; well D: NisA-H_6_ T2D P9D K12D N20E with four negatively charged residues; well E: NisA-H_6_ T2D P9D K12D N20E H27D K34E with six negatively charged residues. The presence of the halo indicates the presence of active nisin processed by NisP to remove the leader.

Introduction of negatively charged residues at different positions of the core peptide part of precursor nisin results in a severe decrease in antimicrobial activity (Fig. 3). The notion that the backbones of rings A and B interact with lipid II [13] and that the C-terminal part of nisin is responsible for the interactions with the lipid membrane [35] allows us to classify the substitutions into categories: the T2D- lipid II interactions, the P9D- lipid II interactions, the K12D- membrane interactions, the N20E- membrane interactions, the H27D- membrane interactions, the K34E- membrane interactions. The general trend that is observed constitutes a severe decrease in the antimicrobial potency of nisin with increasing numbers of negatively charged residues introduced into the core peptide part.

## Discussion

The substrate specificity of lantibiotic modification enzymes is not yet well understood, although some rules for the posttranslational modifications have been proposed [32]. Despite many attempts, obtaining the crystal structure of NisB is still a challenge. However, recently the long-awaited *in vitro* activity of NisB has been successfully reconstituted [36] which will lead to a more detailed understanding of the mechanism of NisB. In order to shed more light on the substrate specificities of this interesting enzyme, we made specific mutations in the core peptide part of precursor nisin and analyzed these variants a) for binding with the nisin modification enzymes NisB and NisC, b) for their antimicrobial activity in case of negative charge altered mutants.

Although serines and threonines are the residues that NisB specifically modifies, here we demonstrate that precursor nisin lacking all serines and threonines in the core peptide part still binds to and can co-purify NisB and NisC in the precursor nisin-modification enzyme pull-down assay [5]. This suggests that the serines and threonines are not involved in direct binding to NisB and strengthens the notion that the leader peptide in particular has the strongest contribution to modification enzyme binding. Notably, the Cys-less precursor nisin pulls down both NisC and the NisC-NisA complex, whereas the Ser/Thr-less precursor pulls down only NisC. This may indicate the importance of cysteines in precursor nisin for a release of NisA from NisC, i.e. the absence of cysteines may lead to incomplete reactions and thus inefficient release of NisA from NisC.

Our study demonstrates that the presence of increasing numbers of negatively charged residues in the core peptide part does not lead to decreased NisB-precursor nisin interactions either. Notably, although negatively charged residues in the core peptide part do not interfere with the binding of precursor nisin mutants to the modification enzymes NisB and NisC, NisA-H_6_ T2D P9E, NisA-H_6_ T2D P9E K12D N20E and NisA-H_6_ T2D P9E K12D N20E H27D K34E mutants have strongly decreased antimicrobial activity levels, demonstrating that the negatively charged residues at positions T2, P9, K12, N20, H27 and K34 have an adverse effect on the antimicrobial activity of nisin. However, the observed antimicrobial activity suggests the presence of at least three lanthionine rings and indicates that these mutants are partially dehydrated.

The C-terminal region of nisin is responsible for interactions with the target membrane, in particular with negatively charged lipids [35]. The nisin rings A and B are responsible for the interactions with the pyrophosphates of lipid II [12], [13]. Depending on the position, the decreased levels of the antimicrobial activity of the nisin mutants containing negatively charged residues in the core peptide part are probably due to a) decreased affinity for the pyrophosphate moiety of lipid II (positions T2, P9), b) decreased affinity for negatively charged phospholipids (positions K12, N20, H27 and K34) and c) a lack of one or more lanthionine rings. The first two thioether rings in nisin, i.e. ring A and B (Fig. 1) form a cage-like structure that interacts with the electronegative pyrophosphate moiety of lipid II. The negatively charged residues introduced in the N-terminal part of nisin, i.e. T2D P9D K12D N20E, are highly likely to be unfavourable in terms of electrostatic interactions with lipid II. Changing T to D at position 2 of nisin results in a more bulky side chain. Most likely, in addition to the unfavourable electrostatic effect, this leads to steric hindrance of the altered cage-like structure with the pyrophosphate moiety. The steric hindrance effect is also supported by the notion that mutation of the adjacent residue, i.e. S3T leads to 12 fold-reduced antimicrobial activity [30]. The Ser at position 3 is in the D-configuration, and the decrease in the antimicrobial activity by changing the thioether ring A from lanthionine to methyllanthionine remained unexplained until NMR studies [13] demonstrated that the additional methyl group would point in the space surrounded by the cage structure of the rings. To the best of our knowledge, the T2D substitution has never been studied before, while the T2A, the T2S and the T2V single point nisin mutants did not lead to a significant change in antimicrobial activity [37].

Our results show that the presence of negatively charged residues in the C-terminus of NisA (NisA-H_6_ T2D P9D K12D N20E H27D K34E mutant) further decreases the antimicrobial activity of nisin (Fig. 3). This result is in agreement with previously published data, where the nisin Z V32E mutant, containing an extra negative charge, was analyzed for interactions with negatively charged lipids and for its antimicrobial activity [35]. In this study, NisZ V32E was demonstrated to have a decreased affinity towards negatively charged lipids together with a concomitant decreased antimicrobial activity [35]. Our study demonstrates a general trend that the increase in negative charges in the nisin core part lowers the antimicrobial activity of the mutants. With a few negative charges there should be at least three lanthionine rings present because mutants with rings A, B and C correctly formed are known to retain some activity. In case of high numbers of negative charges we cannot exclude that multiple lack of modification, as also suggested by Rink et al [32], is also responsible for the negligible activity observed. However, the substrate peptides can still be bound by NisBC. The presence of negatively charged residues in the core peptide part of precursor nisin lead to severely decreased antimicrobial activity of the nisin mutants bearing negatively charged residues, either caused by incomplete modification reactions or reduced interactions with lipid II and/or membranes or both. Serines/threonines and cysteines are not necessary for the interactions of the precursor nisin core peptide with the nisin modification enzymes. These findings can guide further engineering of lantibiotics as a potential class of alternative antibiotics.
