# Correction: The Presence of Modifiable Residues in the Core Peptide Part of Precursor Nisin Is Not Crucial for Precursor Nisin Interactions with NisB- and NisC

**DOI:** 10.1371/journal.pone.0095946

**Published:** 2014-04-23

**Authors:** Rustem Khusainov, Oscar P. Kuipers

Due to an error during the production stages, Figure 2C in the article incorrectly displayed Figure 2C from our publication below:

FEBS Open Bio. 2013 May 30;3:237-42. doi: 10.1016/j.fob.2013.05.001. Identification of distinct nisin leader peptide regions that determine interactions with the modification enzymes NisB and NisC. Khusainov R, Moll GN, Kuipers OP

We are providing a corrected Figure 2 and the raw data for this figure.

The original image displayed as Figure 2C is licensed by *FEBS Open Bio* under a Creative Commons BY-NC-ND license. *PLOS ONE* requires that all content is published under the Creative Commons Attribution License (CC BY) and as a result, we have removed the previous Figure 2C from this article and replaced it by the corrected figure.

## Supporting Information

File S1 Raw data for Figure 2
