# Supplementary figures and images for: Correction: The Presence of Modifiable Residues in the Core Peptide Part of Precursor Nisin Is Not Crucial for Precursor Nisin Interactions with NisB- and NisC
Source: PLoS One. 2014 Apr 23;9(4):e95946. doi: 10.1371/journal.pone.0095946 (PMC3997513; doi:10.1371/journal.pone.0095946)

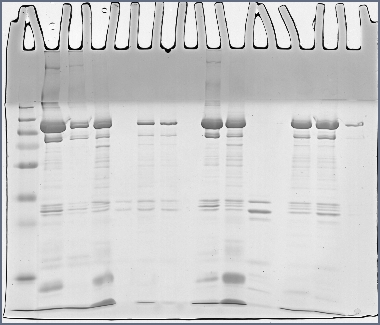


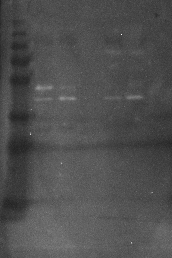

Supplement: File S1 — Raw data for Figure 2 [file pone.0095946.s001.docx]
